# Intermolecular Interactions and Charge Resonance Contributions to Triplet and Singlet Exciton States of Oligoacene Aggregates

**DOI:** 10.3390/molecules28010119

**Published:** 2022-12-23

**Authors:** Yasi Dai, Alessandro Calzolari, Maria Zubiria-Ulacia, David Casanova, Fabrizia Negri

**Affiliations:** 1 Dipartimento di Chimica “Giacomo Ciamician”, Università di Bologna, 40126 Bologna, Italy; 2 Donostia International Physics Center (DIPC), 20018 Donostia-San Sebastian, Euskadi, Spain; 3 Kimika Fakultatea, Euskal Herriko Unibertsitatea (UPV/EHU), Manuel Lardizabal 3, 20018 Donostia-San Sebastian, Euskadi, Spain; 4 IKERBASQUE, Basque Foundation for Science, 48009 Bilbao, Euskadi, Spain; 5 INSTM UdR Bologna, 40126 Bologna, Italy

**Keywords:** oligoacenes, exciton states, singlet states, triplet states, TDDFT, diabatization, adiabatic states, diabatic states, frenkel excitons, charge resonance states, charge transfer states

## Abstract

Intermolecular interactions modulate the electro-optical properties of molecular materials and the nature of low-lying exciton states. Molecular materials composed by oligoacenes are extensively investigated for their semiconducting and optoelectronic properties. Here, we analyze the exciton states derived from time-dependent density functional theory (TDDFT) calculations for two oligoacene model aggregates: naphthalene and anthracene dimers. To unravel the role of inter-molecular interactions, a set of diabatic states is selected, chosen to coincide with local (*LE*) and charge-transfer (*CT*) excitations within a restricted orbital space including two occupied and two unoccupied orbitals for each molecular monomer. We study energy profiles and disentangle inter-state couplings to disclose the (*CT*) character of singlet and triplet exciton states and assess the influence of inter-molecular orientation by displacing one molecule with respect to the other along the longitudinal translation coordinate. The analysis shows that (*CT*) contributions are relevant, although comparably less effective for triplet excitons, and induce a non-negligible mixed character to the low-lying exciton states for eclipsed monomers and for small translational displacements. Such (*CT*) contributions govern the *L_a_/L_b_* state inversion occurring for the low-lying singlet exciton states of naphthalene dimer and contribute to the switch from H- to J-aggregate type of the strongly allowed *B_b_* transition of both oligoacene aggregates.

## 1. Introduction

Electronic and electro-optical materials based on organic molecules have been the subject of numerous investigations ranging from basic materials science to possible technological applications [1,2,3]. Oligoacenes have attracted great interest for energy and charge transport and have emerged as model organic systems for low cost, flexible, large-scale optoelectronic devices [4,5]. The properties of such devices strongly depend on the photophysical behavior of the organic molecular material, ultimately governed by the nature of their low-lying exciton states [6,7,8,9,10,11,12,13,14,15,16,17,18,19,20,21,22]. Thus, to exploit the electro-optical properties of organic semiconductors and to tune device performances, it is desirable to fully understand the character of their electronic excitations.

Although the assessment of the nature of singlet exciton states in crystals and aggregates of organic chromophores has been the subject of several studies [23,24,25,26,27,28,29,30,31,32,33,34], the low-lying triplet exciton states have been addressed in comparably fewer investigations [35,36,37,38,39,40,41,42,43], even though triplet excitons play a fundamental role in several photoinduced processes. For instance, they are often responsible for detrimental non-radiative charge recombination in organic photovoltaic devices [44] or can be relevant for application in photocatalysis and photodynamic therapy [45,46,47].

The exciton states of molecular materials are superpositions of local (intra-molecular) excitations (*LEs*) and charge transfer (*CT*) (i.e., inter-molecular) excitations. Such intra- and inter-molecular excitations can be considered as a suitable basis of diabatic states describing electron promotions between occupied molecular orbitals to unoccupied molecular orbitals of the same (neutral) or neighboring (ionic) molecules (or sites), respectively [26,34,36,48,49,50,51,52,53,54,55,56,57,58,59]. 

Numerous investigations have underscored the relevance of *CT* states in several photoinduced processes: for example, they favor intersystem crossing through spin orbit coupling [60] or mediate singlet fission [61,62,63,64,65,66,67]. *CT* states have also a crucial role in the formation of excimers [37,39,42] and in exciton-dissociation and charge-separation in hetero- and homo-junctions [68,69,70]. 

To analyze the character of excitonic states predicted by quantum-chemical (QC) calculations, a diabatization procedure can be used to determine the superposition of *LE* and *CT* diabatic states in each adiabatic exciton state [26,29,36,55,57]. Recently, a simple diabatization approach has been developed and applied to disentangle the nature of exciton states of perylene di-imide (PDI) aggregates computed with time dependent density functional theory (TDDFT) [30,38].

**Figure 1:**
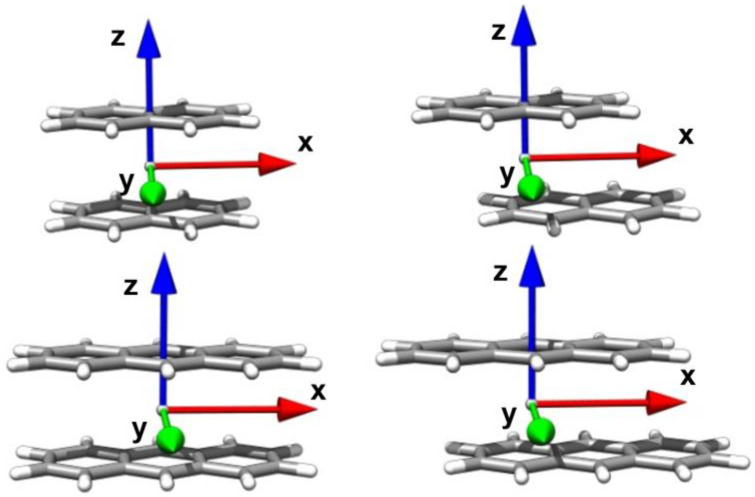
The oligoacene dimers considered in this work. Singlet and triplet exciton states have been determined at the eclipsed configuration and along the interchromophore longitudinal (*x* axis) translation coordinate.

In the present work, we seek to provide a better understanding of the photophysical properties of two oligoacene aggregates, naphthalene and anthracene, and employ the same diabatization procedure to characterize singlet and triplet exciton states in terms of *LE* and *CT* contributions. The approach is however extended to include a larger orbital space, required to correctly describe the orbital nature of the low-lying oligoacene excited states. Oligoacene dimers in their triplet and singlet exciton states are perfect model systems for the investigation and understanding of triplet-triplet and singlet-singlet interactions between π-conjugated molecules, and how these interactions are modulated by different intermolecular organizations. Specifically, here we focus on the longitudinal translation coordinate (Figure 1).

The paper is organized as follows: first we discuss the selection of the orbital space, mandatory to correctly define the diabatic basis for the lowest energy exciton states of oligoacene aggregates. Within the chosen orbital space, we perform a diabatization of singlet and triplet exciton states and discuss their nature, with specific attention to the magnitude of *CT* contributions and, for the LE components, to the parentage with isolated molecule low-lying excited states. The results of our protocol are compared with those obtained through the decomposition of the one-electron transition density matrix performed with the TheoDORE package [71]. Finally, the modulation of the absorption spectrum of oligoacene aggregates along the longitudinal translation coordinate is discussed.

## 2. Results and Discussion

### 2.1. Orbital Space and Diabatic States for the Analysis of Exciton States

**Figure 2:**
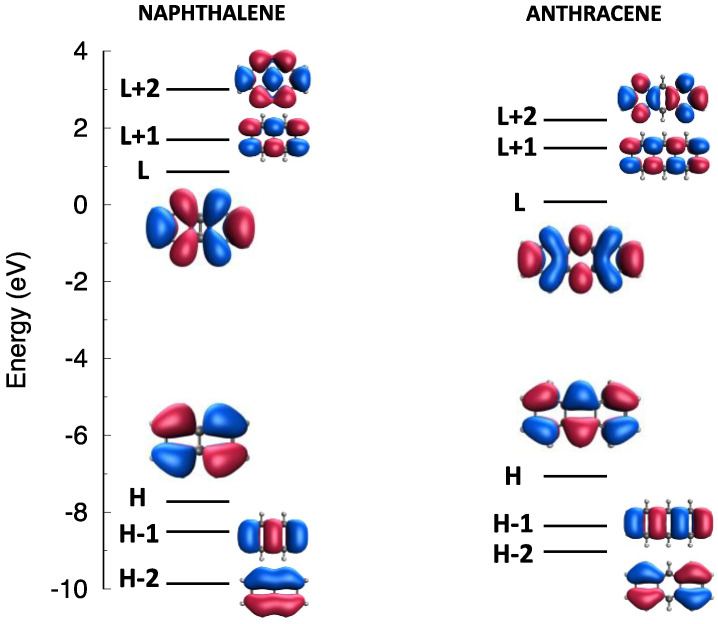
Frontier molecular orbital levels and shapes of (**left**) naphthalene and (**right**) anthracene, from ωB97X-D/6-31G* calculations (HOMO and LUMO abbreviated as H and L).

The most relevant frontier molecular orbitals (MOs) of naphthalene and anthracene are collected in Figure 2, while the lowest excited states of triplet and singlet spin multiplicity (computed with TDDFT calculations in the Tamm-Dancoff approximation (TDA) [72]) for the isolated monomers, are collected in Tables S1 and S2. As it is well known according to the perimeter model [73], the lowest lying excited states of oligoacenes [73,74,75] are determined by excitations within an orbital space that includes at least two occupied (HOMO and HOMO-1) and two unoccupied (LUMO and LUMO+1) MOs leading to four excited states known as the $L_{a},\;L_{b},\;B_{a},B_{b}$ in Platt’s notation [73]. Tables S1 and S2 show that the chosen functional (ωB97X-D, see Section 3) correctly predicts the $S_{1}$ state of naphthalene to be the $L_{b}$ state ($B_{2u}$ symmetry, dominated by a combination of the HOMO-1→LUMO and HOMO→LUMO+1 excitations), while in the case of anthracene the lowest state is $L_{a}$ ($B_{1u}$ symmetry, dominated by the HOMO→LUMO excitation). Computed excitation energies are generally overestimated, as is typical of the long-range corrected functional employed and of the TDA [76,77]. 

Notably, in between the four $L_{a},\;L_{b},\;B_{a},\;B_{b}$ excited states, there are states originated by excitations also involving the HOMO-2 and the LUMO+2 orbitals. However, these states are relatively high in energy and their omission in the following discussion of exciton states will not affect the analysis of intermolecular interactions leading to the lowest exciton states of the two oligoacenes. Accordingly, the orbital space selected in the following analysis of exciton states includes two unoccupied and two occupied MOs for each monomer and, consequently, four occupied and four unoccupied orbitals for their dimers. Due to inter-molecular interactions, the energy levels associated to these dimer orbitals show distinctive oscillations along the longitudinal translation coordinate (Figures S1 and S2). The number of spin-adapted singly excited configurations (and states) originated by an orbital space of n doubly occupied and n unoccupied orbitals is $n^{2}$, such that sixteen exciton states have been considered for each spin multiplicity and their wavefunctions expressed in terms of diabatic states, with the diabatization procedure outlined in Section 3.

The protocol employed to analyze the intermolecular interactions leading ultimately to the character of exciton states, transforms the amplitudes of TDDFT calculations carried out on the aggregate, from the basis of single excitations between aggregate’s orbitals (the delocalized excitation (DE) basis) to the basis of single excitations between molecular (monomers A and B) site orbitals. Localized orbitals on monomers A and B allow to define diabatic states of four different types as follows:

(1) $$LE_{A}^{\left(1\right)}=|1_{A}〉={\left(H-1\right)}_{A}\to {\left(L+1\right)}_{A}\;LE_{B}^{\left(1\right)}=|1_{B}〉={\left(H-1\right)}_{B}\to {\left(L+1\right)}_{B}\;CT_{AB}^{\left(1\right)}=|1_{AB}〉={\left(H-1\right)}_{A}\to {\left(L+1\right)}_{B}\;CT_{BA}^{\left(1\right)}=|1_{BA}〉={\left(H-1\right)}_{B}\to {\left(L+1\right)}_{A}\;\;LE_{A}^{\left(2\right)}=|2_{A}〉={\left(H-1\right)}_{A}\to {\left(L\right)}_{A}\;LE_{B}^{\left(2\right)}=|2_{B}〉={\left(H-1\right)}_{B}\to {\left(L\right)}_{B}\;CT_{AB}^{\left(2\right)}=|2_{AB}〉={\left(H-1\right)}_{A}\to {\left(L\right)}_{B}\;CT_{BA}^{\left(2\right)}=|2_{BA}〉={\left(H-1\right)}_{B}\to {\left(L\right)}_{A}\;LE_{A}^{\left(3\right)}=|3_{A}〉={\left(H\right)}_{A}\to {\left(L\right)}_{A}\;LE_{B}^{\left(3\right)}=|3_{B}〉={\left(H\right)}_{B}\to {\left(L\right)}_{B}\;CT_{AB}^{\left(3\right)}=|3_{AB}〉={\left(H\right)}_{A}\to {\left(L\right)}_{B}\;CT_{BA}^{\left(3\right)}=|3_{BA}〉={\left(H\right)}_{B}\to {\left(L\right)}_{A}\;LE_{A}^{\left(4\right)}=|4_{A}〉={\left(H\right)}_{A}\to {\left(L+1\right)}_{A}\;LE_{B}^{\left(4\right)}=|4_{B}〉={\left(H\right)}_{B}\to {\left(L+1\right)}_{B}\;CT_{AB}^{\left(4\right)}=|4_{AB}〉={\left(H\right)}_{A}\to {\left(L+1\right)}_{B}\;CT_{BA}^{\left(4\right)}=|4_{BA}〉={\left(H\right)}_{B}\to {\left(L+1\right)}_{A}$$

where HOMO and LUMO are abbreviated as H and L, subscripts A and B indicate different monomers, and each type of excitation is differentiated by a superscript.

The diabatization procedure discussed in Section 3 provides the Hamiltonian matrix in the diabatic basis representation, $H_{dia}$. In this work, compared to previous investigations in which only the HOMO and LUMO orbitals were included for each monomer, [29,30,36,38] the $H_{dia}$ matrix (dimension 16 × 16) includes not only interactions between diabatic states of the same type ($LE_{A}^{\left(n\right)},LE_{B}^{\left(n\right)},CT_{AB}^{\left(n\right)},CT_{BA}^{\left(n\right)}$), such as excitonic interactions $V_{e}^{\left(n\right)}$ and super-exchange interactions [78] $D_{e/h}^{\left(n\right)}$ (Table S3), but also interactions between diabatic states of different type ($LE/CT_{}^{\left(n\right)},LE/CT_{}^{\left(p\right)}$), hereafter labeled with two superscript numbers, e.g., $V_{e}^{\left(n,p\right)}$ (Table S4). In addition, intramolecular interactions between diabatic states localized on the same monomer ($LE_{A}^{\left(n\right)},LE_{A}^{\left(p\right)}$) are also uncovered by the diabatization procedure ($H^{\left(n,p\right)}$ in Table S4).

For aggregates characterized by a symmetric arrangement of chromophores, as is the case for those investigated here, when the molecules approach each other, intermolecular interactions mix the LE states to form a symmetry adapted (SA) superposition of (neutral) LE states, that is, Frenkel excitons (FE). Similarly, CT states form delocalized charge resonance (CR) states [48,79,80] of appropriate symmetry. The symmetry point group of an oligoacene aggregate, when intermolecular displacements along the longitudinal translation coordinate are considered, (Figure 1) is $C_{2h}$. As a result, the most relevant $\pi \pi^{*}$ exciton states along with FE and CR diabatic states, all belong to $A_{g},A_{u},\;B_{g}$ and $B_{u}$ symmetry representations. In the following Figures, each symmetry will be distinguished by a specific color code used throughout this work: yellow for $A_{g}$, orange for $A_{u}$, blue for $B_{g}$ and green for $B_{u}$.

**Figure 3:**
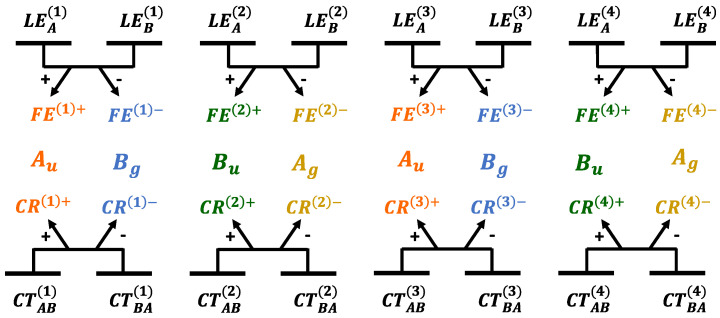
Definition of SA diabatic states (FE, CR, each symmetry with a specific color code used throughout this work: yellow for $A_{g}$, orange for $A_{u}$, blue for $B_{g}$ and green for $B_{u}$) defined as linear combinations of diabatic states (black, LE, CT) and employed to analyze the nature and the effect of interactions on the adiabatic exciton states of oligoacene dimers.

The SA diabatic states are then obtained as linear combinations of LE and CT states as shown in Figure 3 and the corresponding diabatic matrices $H_{dia}^{SA}$ are collected in Tables S5 and S6. The energy profiles of the diabatic states and the most relevant interactions extracted from the diabatization procedure are collected in Figures S3–S9.

In the following sections we analyze the TDA computed singlet and triplet exciton states of oligoacene dimers to determine their character (CT/LE) and disentangle the role of interactions between diabatic states along the longitudinal translation coordinate.

### 2.2. Singlet Exciton States of Naphthalene and Anthracene Dimers

**Figure 4:**
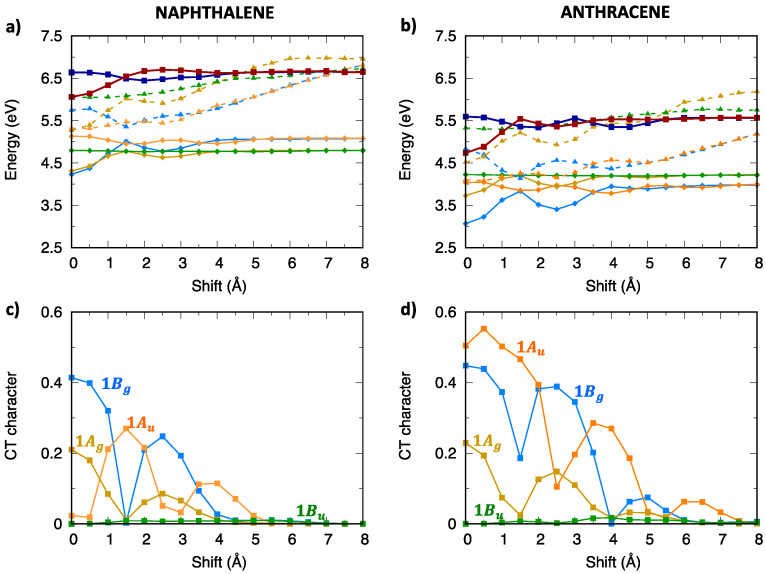
Lowest energy singlet exciton states of (**left**) naphthalene and (**right**) anthracene: (**a**,**b**) adiabatic excitation energy profiles of the low-lying exciton states depicted with different color codes for different symmetries: yellow for $A_{g}$, orange for $A_{u}$, blue for $B_{g}$ and green for $B_{u}$. The lowest energy exciton states not included in the diabatization procedure are also shown (dark blue, $B_{g}$ symmetry, dark red, $A_{u}$ symmetry), (**c**,**d**) CT character of the lowest four exciton states.

The TDA computed excitation energy profiles of the singlet exciton states of naphthalene and anthracene dimers are collected in Figure 4 and Figure S10. We note that $B_{1u}$ ($L_{a}$) excited states of the isolated molecules originate two LE states in the dimer, belonging to $A_{u}$ and $B_{g}$ symmetries of the $C_{2h}$ point group, while $B_{2u}$ ($L_{b}$) monomer excited states originate two LE states in the dimer belonging to $A_{g}$ and $B_{u}$ symmetries. Because the lowest excited states of naphthalene and anthracene are, respectively, $L_{b}$ and $L_{a}$, one might expect the lowest exciton state with different nature and symmetry for the two aggregates. In contrast, both naphthalene and anthracene dimers show that, for small displacements from the eclipsed geometry, the lowest energy exciton state belongs to $B_{g}$ symmetry, which therefore originates from the $L_{a}$ ($B_{1u}$) monomer state for both dimers. This result is expected for anthracene while it implies an inversion of the $L_{b}/L_{a}$ states for naphthalene, when moving from the monomer to the aggregate. Such an inversion has been documented in previous studies at the equilibrium intermolecular distance of the singlet state excimer [81,82], which is considerably shorter than the intermolecular distance of 3.4 Å considered here. Interestingly, the stabilization of the lowest $B_{g}$ state leading to the formation of the naphthalene excimer is predicted by our TDA-ωB97X-D calculations even at such large intermolecular distances.

**Figure 5:**
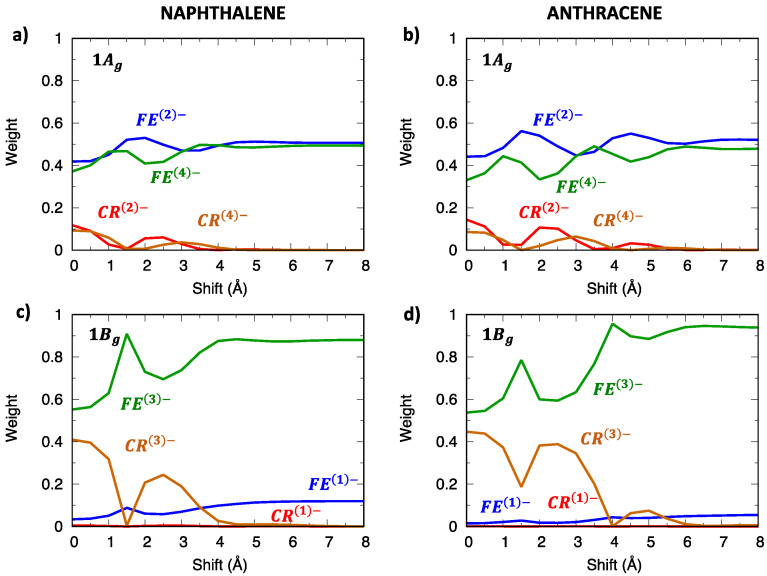
Wavefunction composition of the adiabatic lowest exciton states of (**left**) naphthalene and (**right**) anthracene in terms of the SA diabatic states defined in Figure 3. (**a**,**b**) $1A_{g}$ state and (**c**,**d**) $1B_{g}$ state. Red and brown lines represent the contributions to the total wavefunction of CT states, while green and blue lines represent the weight of LE states.

Although the 1$B_{g}$ exciton state of the oligoacene dimers derives from the $L_{a}$ state and therefore owns a considerable LE contribution, its character is indeed mixed, showing more that 40% CT contribution (Figure 4c,d) at the eclipsed geometry. This is true not only for the 1$B_{g}$ state, but also for other low-lying exciton states, with the CT contribution slightly increasing from naphthalene to anthracene dimers. Such a mixed character can be appreciated by graphically representing the wavefunction of the two lowest exciton states (1$B_{g}$ and 1$A_{g})$ in terms of their SA diabatic states. The contributions of CR states (red and brown lines in Figure 5) emerge clearly not only for the eclipsed dimer configuration, but up to about 4 Å longitudinal displacements, even though the weight of FE states (green and blue lines) is dominant. 

**Figure 6:**
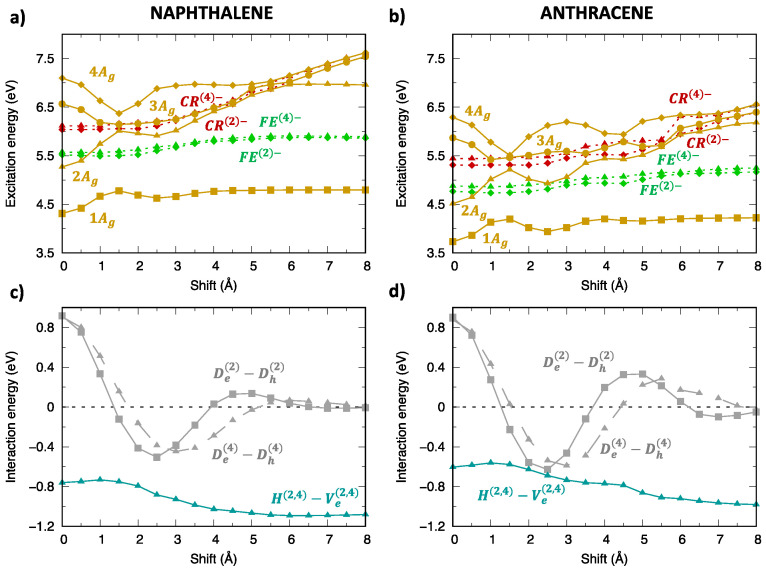
Analysis of the excitation energy profiles of singlet exciton states ($A_{g}$ symmetry) for (**left**) naphthalene and (**right**) anthracene dimer (TDA-ωB97X-D/6-31G*) in terms of SA diabatic states (green for FE states, red for CR states) and their interactions. (**a**,**b**) Computed adiabatic and SA diabatic excitation energy profiles. (**c**,**d**) Magnitude and modulation along the longitudinal translation coordinate of the (grey) $D_{e}^{\left(2\right)}-D_{h}^{\left(2\right)}$, $D_{e}^{\left(4\right)}-D_{h}^{\left(4\right)}$ interactions, coupling FE and CR states, and of the (dark-turquoise) $H^{\left(2,4\right)}-V_{e}^{\left(2,4\right)}$ interactions mixing $FE^{\left(2\right)}$ and $FE^{\left(4\right)}$ states.

**Figure 7:**
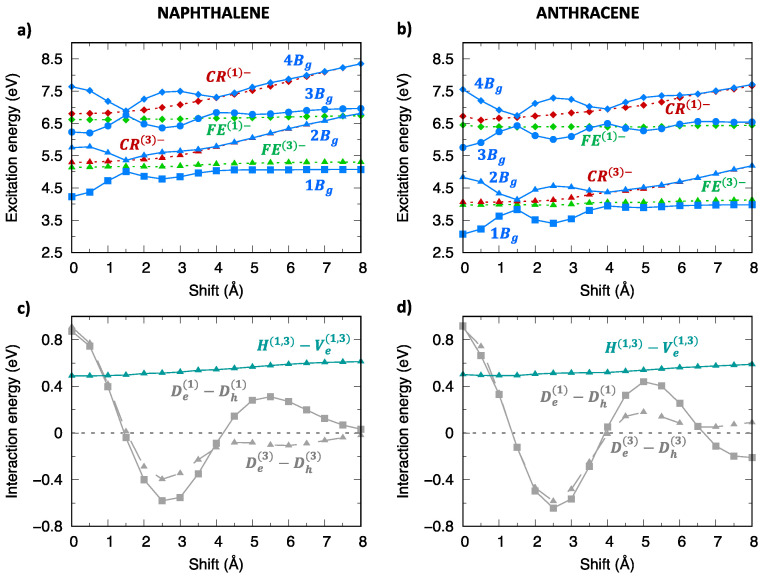
Analysis of the excitation energy profiles of singlet exciton states ($B_{g}$ symmetry) for (**left**) naphthalene and (**right**) anthracene dimer (TDA-ωB97X-D/6-31G*) in terms of SA diabatic states (green for FE states, red for CR states) and their interactions. (**a**,**b**) Computed adiabatic and SA diabatic excitation energy profiles. (**c**,**d**) Magnitude and modulation along the longitudinal translation coordinate of the (grey) $D_{e}^{\left(1\right)}-D_{h}^{\left(1\right)}$, $D_{e}^{\left(3\right)}-D_{h}^{\left(3\right)}$ interactions, coupling FE and CR states, and of the (dark-turquoise) $H^{\left(1,3\right)}-V_{e}^{\left(1,3\right)}$ interactions mixing $FE^{\left(1\right)}$ and $FE^{\left(3\right)}$ states.

As shown in previous investigations [29,30,36,38], the modulation of adiabatic energy profiles along the longitudinal translation coordinate can be rationalized in terms of inter-state interactions between SA diabatic states. The different effects of inter-state and intermolecular interactions can be appreciated by comparing SA diabatic and resulting adiabatic energy profiles of $A_{g}$ (Figure 6) and $B_{g}$ (Figure 7) symmetry. In both cases, the adiabatic exciton energy profiles result from the combination of i) the interaction between FE and CR SA diabatic states (grey lines in panels (c,d)), which is maximum at the eclipsed geometry and oscillates along the translation, with ii) the interaction between the two FE SA diabatic states (dark-turquoise lines in panels (c,d)), less dramatically changing along the translational coordinate. Such an interaction is larger for $A_{g}$ symmetry states and more effective, given the quasi degeneracy of $FE^{\left(2\right)-}$ and $FE^{\left(4\right)-}$ energy profiles, while it is far less effective for $B_{g}$ states due to the large energy difference between $FE^{\left(1\right)-}$ and $FE^{\left(3\right)-}$ states. This explains the remarkable energy lowering of the $1A_{g}$ adiabatic exciton state (yellow squares in Figure 6a,b) compared to the SA diabatic states (green) for all translational displacements, which is not observed for $B_{g}$ states except for specific displacement ranges corresponding to large FE/CR interactions. Similar considerations justify the excitation energy profiles of adiabatic exciton states belonging to the remaining two symmetries (Figures S11 and S12).

### 2.3. Triplet Exciton States of Naphthalene and Anthracene Dimers

**Figure 8:**
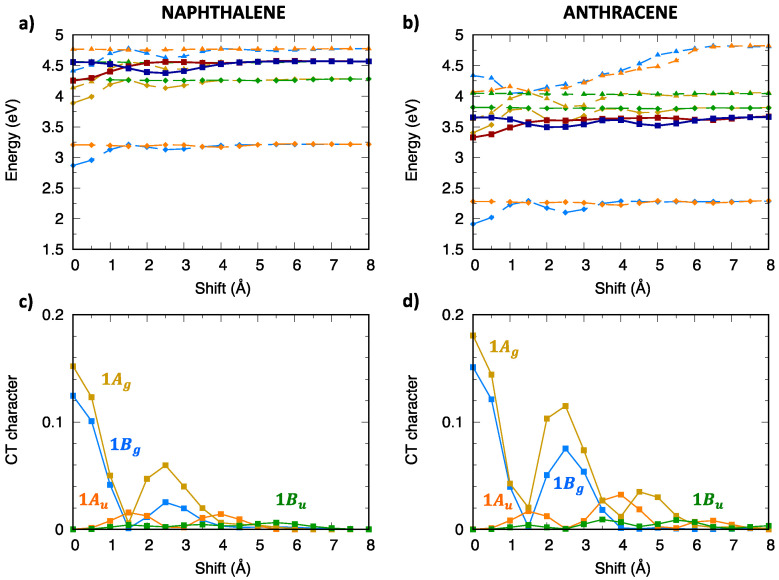
Lowest energy triplet exciton states of (**left**) naphthalene and (**right**) anthracene: (**a**,**b**) adiabatic excitation energy profiles of the low-lying exciton states depicted with different color codes for different symmetries: yellow for $A_{g}$, orange for $A_{u}$, blue for $B_{g}$ and green for $B_{u}$. The lowest energy exciton states not included in the diabatization procedure are also shown (dark blue, $B_{g}$ symmetry, dark red, $A_{u}$ symmetry); (**c**,**d**) CT character of the lowest four exciton states.

Figure 8 and Figure S10 collect the excitation energy profiles of triplet excitons of oligoacene aggregates. In contrast with singlet excitons, we note that the lowest two triplet exciton states belong to $B_{g}$ and $A_{u}$ symmetries, while the lowest $A_{g}$ exciton state is found at much higher energy. Similar to singlet excitons, a few low-lying states (dark-blue/dark-red in Figure 8) were not included in the diabatization procedure, since their influence is negligible due to their different orbital nature and energy separation from the lowest exciton states. The CT character analysis (Figure 8c,d) demonstrates that both the 1$B_{g}$ and $1A_{g}$ states have a mixed CT/LE character with the CT contribution slightly increasing for the longer acene dimer.

**Figure 9:**
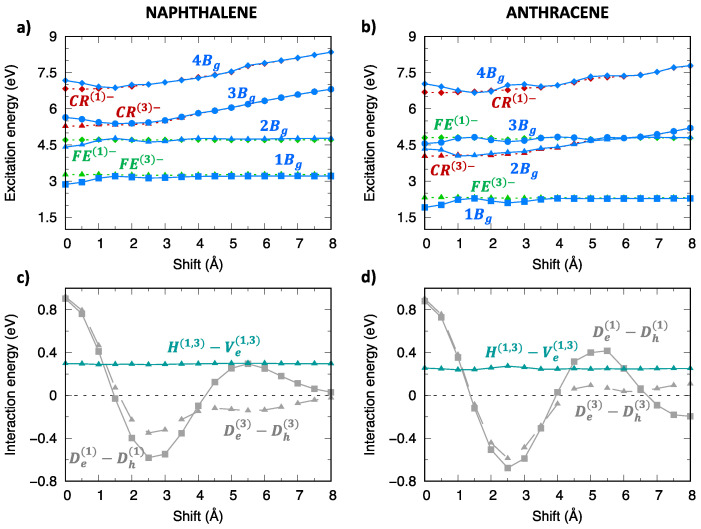
Analysis of the excitation energy profiles of triplet exciton states ($B_{g}$ symmetry) for (**left**) naphthalene and (**right**) anthracene dimer (TDA-ωB97X-D/6-31G*) in terms of SA diabatic states (green for FE states, red for CR states) and their interactions. (**a**,**b**) Computed adiabatic and SA diabatic excitation energy profiles. (**c**,**d**) Magnitude and modulation along the longitudinal translation coordinate of the (grey) $D_{e}^{\left(1\right)}-D_{h}^{\left(1\right)}$, $D_{e}^{\left(3\right)}-D_{h}^{\left(3\right)}$ interactions, coupling FE and CR states, and of the (dark-turquoise) $H^{\left(1,3\right)}-V_{e}^{\left(1,3\right)}$ interactions mixing $FE^{\left(1\right)}$ and $FE^{\left(3\right)}$ states.

**Figure 10:**
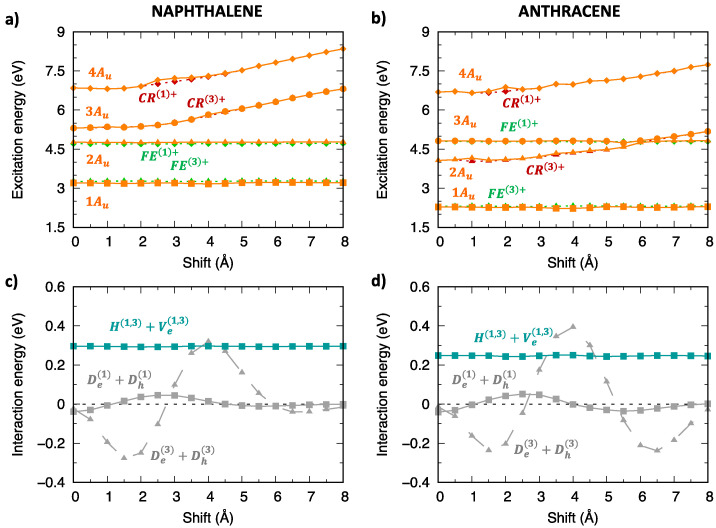
Analysis of the excitation energy profiles of triplet exciton states ($A_{u}$ symmetry) for (**left**) naphthalene and (**right**) anthracene dimer (TDA-ωB97X-D/6-31G*) in terms of SA diabatic states (green for FE states, red for CR states) and their interactions. (**a**,**b**) Computed adiabatic and SA diabatic excitation energy profiles. (**c**,**d**) Magnitude and modulation along the longitudinal translation coordinate of the (grey) $D_{e}^{\left(1\right)}+D_{h}^{\left(1\right)}$, $D_{e}^{\left(3\right)}+D_{h}^{\left(3\right)}$ interactions, coupling FE and CR states, and of the (dark-turquoise) $H^{\left(1,3\right)}+V_{e}^{\left(1,3\right)}$ interactions mixing $FE^{\left(1\right)}$ and $FE^{\left(3\right)}$ states.

The significant CT contribution to 1$B_{g}$ and $1A_{g}$ states, contrasts with the almost negligible contribution to 1$B_{u}$ and $1A_{u}$ states. Such differences can be rationalized by comparing, for instance, the SA diabatic energy profiles ($B_{g}$ and $A_{u}$ symmetry) with FE and CR character (green and red curves, respectively, in Figure 9 and Figure 10) and their inter-state interactions, depicted in the bottom part of the above figures. Similar considerations hold for the other symmetry species (Figures S13 and S14). The FE/CR energy differences are very similar for the two sets of SA diabatic states, but their couplings (grey lines) are much larger for the $B_{g}$ symmetry states. Specifically, the largest interaction amounts to more than 0.8 eV for $B_{g}$ and it does not exceed 0.3 eV for $A_{u}$ states. As a result, $1B_{g}$ adiabatic states display a non-negligible CT character for small longitudinal displacements, while $1A_{u}$ states do not. 

The analysis of interactions between SA diabatic states uncovers also specific differences between singlet and triplet excitons. One well-known distinctive element, previously documented for PDI aggregates [36,38], is the larger energy separation between CR and FE triplet diabatic states compared to singlet states, accounting for the reduced effect of super-exchange interactions, ultimately leading to a less marked CT character of lowest-lying triplet exciton states. An additional distinction can be appreciated in the case of oligoacenes and concerns the couplings (Tables S5 and S6) between SA diabatic states of FE type (dark-turquoise lines in Figure 6 and Figure 7 for singlet and Figure 9 and Figure 10 for triplet excitons). These are larger than 0.8 eV for singlet SA states of $A_{g}/B_{u}$ symmetry while they do not exceed 0.35 eV for the triplet SA states of any symmetry. As a result, the adiabatic triplet exciton states of all symmetry species almost overlap with the SA diabatic states for large portions of the energy profiles, in contrast with those of singlet spin multiplicity discussed in the previous section. These interactions result from the combination of an intra-molecular contribution (top part of Figures S8 and S9) with the inter-molecular exciton coupling (bottom part of Figures S8 and S9) both of which are much larger for singlet states.

### 2.4. CT Character of Singlet and Triplet Excitons of Oligoacenes

**Figure 11:**
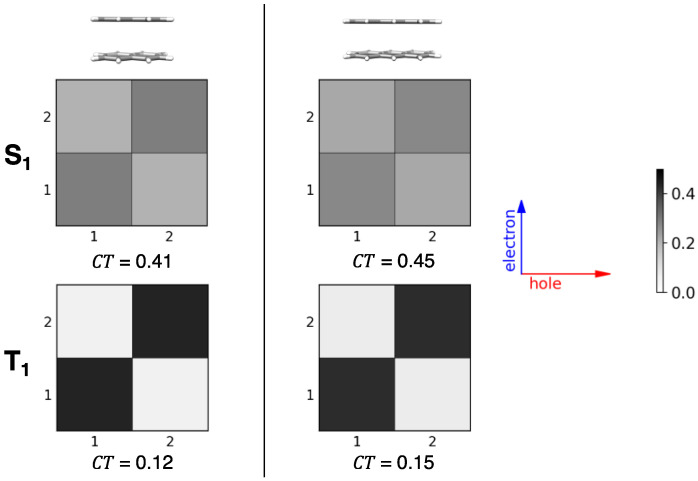
Lowest energy singlet and triplet exciton-state analysis via electron-hole correlation plots [71] for naphthalene and anthracene dimers in their eclipsed configuration. The grey scale used is shown on the right panel. From TDA-ωB97X-D/6-31G* calculations. The magnitude of the CT contribution is shown on the bottom part of each panel.

The CT character analysis obtained from the above discussed diabatization procedure (Figure 4c,d and Figure 8c,d) can be compared with the results of other analysis tools. To visualize the nature of the lowest singlet and triplet exciton states, for the eclipsed dimer configurations of the two oligoacene dimers, we carried out a fragment-based analysis via electron–hole correlation plots, using TheoDORE [71] (Figure 11). The two selected fragments correspond to the two molecules forming the dimer. Exciton states are identified by the non-vanishing elements of the 2 × 2 matrix (the Ω-matrix [71]) represented by different levels of grey. Locally excited contributions appear in Figure 11 off-diagonally (going from lower left to upper right), while CT contributions appear on the main diagonal. In agreement with the character analysis shown in Figure 4c,d and Figure 8c,d, also Figure 11 shows that for the eclipsed configuration, the character of the lowest singlet and triplet exciton states is mixed LE/CT as indicated by the light-grey squares in the electron-hole correlation plot. As discussed in previous sections, the CT contribution is larger for singlet exciton states than for triplet exciton states (main diagonal squares for the singlet excitons are dark grey while those of triplet exciton states are light grey) as a result of the larger energy separation between CT and LE diabatic triplet states (compare Figures S3 and S5 or Figures S4 and S6). Overall, both approaches provide the same results for the lowest triplet and singlet exciton states as regard the CT contributions, although our diabatization analysis also provides detailed information on relevant intermolecular and interstate interactions. 

### 2.5. Absorption Spectrum and H-/J- Character Switch along the Longitudinal Translation Coordinate

**Figure 12:**
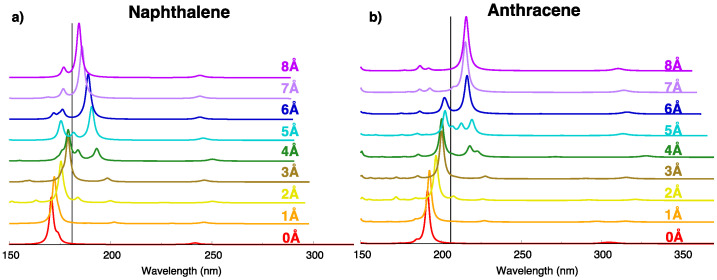
Comparison between vertical absorption spectra predicted for (**a**) naphthalene and (**b**) anthracene dimer, from TDA-ωB97X-D/6-31G* calculations. Modulation along the longitudinal shift from 0 Å to 8 Å. Different colors are used to plot spectra computed for different displacements. Black bar: absorption peak of the $B_{b}$ state of the monomer, calculated at TDA-ωB97X-D/6-31G* level.

In previous sections we focused the attention on low-lying exciton states of the oligoacene dimers. Because the absorption spectrum of oligoacenes is dominated by the transition to the higher energy $B_{2u}$ state (the $B_{b}$ state in Platt’s notation), it is interesting to discuss the evolution (along the displacement coordinate) of the dipole allowed exciton state bearing the largest parentage with the $B_{b}$ state and how its excitation energy modulation influences the appearance of the absorption spectrum. The computed absorption spectrum for increasingly large translational displacements (Figure 12) shows that the intense absorption occurs at higher energies than the isolated molecule, suggesting an H- type aggregate behavior for small displacements from the eclipsed configurations. For larger displacements, the most intense absorption band moves abruptly to lower excitation energies (J- type) after a narrow intermediate region in which two bands of similar intensity appear. The evolution of the excitation energy of such strongly allowed exciton state is shown in black in Figure S15. This state corresponds to the $4B_{u}$ state for both naphthalene and anthracene at the eclipsed geometry and for displacements up to 4 or 5 Å, then it switches to the $2B_{u}$ state. Such a switch is associated with a sudden change in the exciton state character, acquiring a dominant CT contribution for larger displacements (Figure S16), suggesting that the H- to J- type transition along the longitudinal displacement is assisted not only by the sign change of the exciton interaction (Figure S7) but also by the interaction between FE and CR states. 

Finally, for large displacements (8 Å) the strongest absorption peak has moved back almost toward the isolated molecule value, although not completely owing to some still non-negligible exciton interactions. Indeed the $V_{e}^{\left(2\right)}$ and $V_{e}^{\left(4\right)}$ couplings are still not vanishing at such distance (Figure S7), in contrast with the $V_{e}^{\left(1\right)}$ and $V_{e}^{\left(3\right)}$ interactions that have already become negligible. 

## 3. Computational Models

The ground state monomer structure of naphthalene and anthracene was optimized at the ωB97X-D/6-31G* level of theory. The distance between the planes of different monomers was set to 3.4 Å, as used in previous investigations on dimers of other polycyclic aromatic hydrocarbons [29,30]. Exciton states were computed for the eclipsed aggregates and for displacements of 0.5 Å up to 8.0 Å, along the longitudinal translation coordinate (x) (Figure 1). Excitation energies were determined with TDDFT calculations with the TDA [72], using the ωB97X-D functional [83], previously shown to provide a reliable description of CT character in singlet excitons of PDI dimers [30,34,38], and the 6-31G* basis set. All QC calculations were carried out with the Gaussian16 suite of programs [84]. 

To analyze the exciton character, we followed the approach described in previous works [30,38] and we expressed each relevant exciton state in terms of LEs. To this end, we selected the orbital subspace corresponding to relevant $\pi \pi^{*}$ exciton states. As discussed in Section 2, for oligoacene aggregates this must include at least the HOMO/HOMO-1 and LUMO/LUMO+1 of each monomer [27,75] and represents the minimal orbital space (MIOS) sufficient to reliably describe low-lying excited states of each monomer. Each aggregate’s orbital obtained from QC calculations is then expressed as linear combination of monomer orbitals. These linear combination coefficients $C_{i,j}^{AGGR\_MOB}$ form the $C_{AGGR\_MOB}^{}$ matrix describing each aggregate’s orbital in the monomer orbital basis (MOB) and are obtained as [25,30,85]:

(2) $$C_{AGGR\_MOB}^{}=C_{MON\_AOB}^{t}\cdot S_{MON\_AOB}\cdot C_{AGGR\_AOB}\;\;$$

where the $C_{MON\_AOB}$ matrix is a block diagonal matrix containing the MOs coefficients in the atomic orbital basis (AOB) of each monomer, with off-diagonal blocks set to zero and $S_{MON\_AOB}$ is the overlap matrix of the monomers in the AOB. 

Since monomer orbitals belonging to two different molecules are non-orthogonal to each other, aggregate’s orbitals $C_{AGGR\_MOB}^{L}$ expressed in terms of orthogonalized monomer orbitals are obtained as:

(3) $$C_{AGGR\_MOB}^{L}=S_{AGGR\_\_MOB}^{-\frac{1}{2}}\cdot C_{AGGR\_MOB}^{}\;\;$$

where superscript L indicates Löwdin’s orthogonalization [86], and the overlap matrix $S_{AGGR\_MOB}=C_{MON\_AOB}^{t}\cdot S_{AGGR\_AOB}\cdot C_{MON\_AOB}$ is obtained from the coefficients of monomer’s orbitals $C_{MON\_AOB}$ and the overlap of the atomic orbitals in the aggregate configuration $S_{AGGR\_AOB}$.

From the results of TDDFT calculations on the aggregate, the subset of $n^{2}$ exciton states originated from the MIOS of the aggregate (including n occupied and n unoccupied orbitals) are then selected out of the full set of computed eigenstates. TDDFT amplitudes are expressed on the basis of delocalized excitations (DEs), namely excitations between aggregate’s orbitals, and form the columns of the $B_{DE}^{adia}$ matrix. Thus, each DE must be expanded in terms of excitations between monomer orbitals (diabatic LE and CT states). With aggregate’s orbitals expressed in terms of monomer orbitals via the $C_{AGGR\_MOB}^{L}$ matrix, each DE(i→j) from an occupied *i* to an empty *j* aggregate’s orbital can be expressed as a linear combination of diabatic (LE and CT) excitations (k→l) from an occupied *k* to an empty *l* monomer orbital, with expansion coefficients given by

(4) $$U_{k\to l,i\to j}^{DE\to dia}=C_{k,i}^{AGGR\_MOB,\;L}\;\cdot C_{l,j}^{AGGR\_MOB,L}\;$$

these coefficients form the columns of the unitary matrix $U_{DE\;\to dia}$. 

Exciton states are then readily expressed in the diabatic basis as

(5) $$B_{dia}^{adia}=U_{DE\to dia}^{}\cdot B_{DE}^{adia}\;$$

and the character of each exciton state is obtained by summing up the contributions from CT and LE states.

The corresponding $n^{2}$ eigenvalues (excitation energies of the selected adiabatic excitons) form the diagonal $H_{adia}$ matrix, from which the Hamiltonian in the diabatic LE/CT basis, $H_{dia}$, can be obtained as [26,54,57,87]

(6) $$H_{dia}=B_{dia}^{adia}\cdot H_{adia}\cdot B_{dia}^{adia}{}^{t}$$

Finally, the $H_{dia}$ is rotated in the SA diabatic basis formed by FE and CR states, to obtain a block diagonal matrix with four sub-matrices $H_{dia}^{SA}\;$(for $B_{u}$, $A_{g},$ $B_{g}$ and $A_{u}$ states, (Tables S5 and S6)) whose off-diagonal elements are the interactions between CR and FE states that ultimately govern the modulation of adiabatic exciton state energies along the longitudinal translation coordinate. 

## 4. Conclusions

Intermolecular interactions determine the nature of exciton states which ultimately govern the optoelectronic properties and the outcome of photoinduced processes in molecular materials. In this work we have analyzed the character and modulation of singlet and triplet exciton states of two oligoacene homo-dimers formed by naphthalene and anthracene, along the intermolecular longitudinal translation coordinate.

The character of exciton states computed with TDDFT and the relevant inter-molecular and inter-state interactions were determined with a diabatization procedure successfully employed in previous investigations of PDI and here extended to include a larger orbital space, mandatory to correctly describe the low-lying excited states of oligoacenes. 

The analysis in terms of localized excitations shows that CT contributions are relevant, although comparably less effective for triplet excitons, and induce a non-negligible mixed character to the low-lying exciton states for eclipsed monomers and for small translational displacements compatible with the formation of excimers not only for singlet but also for triplet excitons. 

Concerning the singlet spin manifold, the study shows that such CT contributions drive the $L_{a}$/$L_{b}$ state inversion of the lowest-lying exciton state of naphthalene dimer and assist the switch from H- to J-aggregate type of the strongly allowed $B_{b}$ transition of both oligoacene aggregates. 

We believe that this study provides useful insights on the magnitude of inter-molecular interactions occurring in molecular materials and determining the nature of exciton states of both singlet and triplet spin multiplicity, paving the way to future investigations on more complex aggregates and inter-molecular organizations.

## Data Availability

The data presented in this study are available in the Supplementary Material.

## Supplementary Materials

The following supporting information can be downloaded at: https://www.mdpi.com/article/10.3390/molecules28010119/s1, Figure S1. Energy profiles of the frontier molecular orbitals of naphthalene. Figure S2. Energy profiles of the frontier molecular orbitals of anthracene. Figures S3–S6. Energy profiles of the singlet and triplet diabatic exciton states of naphthalene and anthracene. Figure S7. Energy profiles of the excitonic and super-exchange interactions. Figures S8 and S9. Energy profiles of interactions between *LE* diabatic singlet and triplet states. Figure S10. Adiabatic energy profiles of singlet and triplet adiabatic exciton states of naphthalene and anthracene. Figures S11–S14. Analysis of the excitation energy profiles. Figure S15. The evolution of the strongly allowed exciton state of naphthalene and anthracene. Figure S16. CT character of the singlet adiabatic exciton states of Bu symmetry of naphthalene and anthracene. Figure S17. Angle between the transition dipole moments of the monomers forming naphthalene and anthracene dimers. Table S1. Triplet excited states of naphthalene and anthracene monomers. Table S2. Singlet excited states of naphthalene and anthracene monomer. Table S3. Matrix elements of $H_{dia}^{}$ between diabatic states of the same type. Table S4. Matrix elements of $H_{dia}^{}$ between diabatic states of different types. Tables S5 and S6. Matrix elements of $H_{dia}^{SA}$.
