# Supplementary material for: Intermolecular Interactions and Charge Resonance Contributions to Triplet and Singlet Exciton States of Oligoacene Aggregates
Source: Molecules. 2022 Dec 23;28(1):119. doi: 10.3390/molecules28010119 (PMC9822017; doi:10.3390/molecules28010119)
Supplement: Supplementary file 1 [file molecules-28-00119-s001.zip › molecules-2061515-supplementary.pdf]

Supporting Information for

Intermolecular interactions and charge resonance contributions to triplet and singlet exciton states of oligoacene aggregates

Yasi Dai[1], Alessandro Calzolari[1], Maria Zubiria-Ulacia[2,3], David Casanova[2,4] and Fabrizia Negri[1,5]

[1] Dipartimento di Chimica "Giacomo Ciamician", Università di Bologna, 40126 Bologna, Italy

[2] Donostia International Physics Center (DIPC), 20018 Donostia, Euskadi, Spain.

[3] Kimika Fakultatea, Euskal Herriko Unibertsitatea (UPV/EHU), Manuel Lardizabal 3, 20018 Donostia-San Se-bastian, Euskadi, Spain.

[4] IKERBASQUE, Basque Foundation for Science, 48013 Bilbao, Euskadi, Spain.

[5] INSTM UdR Bologna, 40126, Bologna, Italy

Table S1. Excitation energies of the lowest triplet excited states of naphthalene and anthracene monomers. TDA-ωB97X-D /6-31G* calculations. Excited states according to Platt's notation are in bracket. The states out of the chosen monomeric orbital space are in bold type.

		**Naphthalene**			
State	Sym.	Energy (eV)	λ (nm)	Wavefunction	
T1 (L_a)	B1U	3.21	386	0.67	H → L
T2 (L_b)	B2U	4.28	290	0.55	H−1 → L
				−0.42	H → L+1
T3	B3G	4.57	272	0.50	H−2 → L
				0.48	H → L+2
T4 (B_b)	B2U	4.57	271	0.55	H → L+1
				0.43	H−1 → L
T5 (B_a)	B1U	4.77	260	0.68	H−1 → L+1
		**Anthracene**			
State	Sym.	Energy (eV)	λ (nm)	Wavefunction	
T1 (L_a)	B1U	2.29	542	0.68	H → L
**T2**	**B3G**	**3.66**	**339**	**0.50**	**H-2 → L**
				**0.45**	**H → L+2**
T3 (L_b)	B2U	3.83	324	0.61	H−1 → L
				−0.32	H → L+1
T4 (B_b)	B2U	4.05	306	0.61	H → L+1
				0.32	H−1 → L
					H−1 → L+1
**T5**	**B1U**	**4.49**	**276**	**0.44**	**H−3 → L**
				**0.43**	**H → L+3**
				**− 0.32**	**H−2 → L+2**
T6 (B_a)	B1U	4.83	257	0.67	H−1 → L+1

Table S2. Excitation energies of the lowest singlet excited states of Naphthalene and Anthracene monomer. TDA-ωB97X-D /6-31G* calculations. Excited states according to Platt's notation are in bracket. The states out of the chosen monomeric orbital space are in bold type.

			**Naphthalene**			
State	Sym.	Energy (eV)	λ (nm)	Osc.strength	Wavefunction	
S1 (L_b)	B2U	4.80	259	0.0002	0.49	H → L+1
					0.50	H−1 → L
S2 (L_a)	B1U	5.08	244	0.0914	0.65	H → L
S3	B3G	6.65	186	0	0.53	H → L+2
					−0.47	H−2 → L
S4	AG	6.71	185	0	0.47	H−1 → L+2
					0.47	H−2 → L
S5 (B_b)	B2U	6.85	181	1.9677	0.49	H → L+1
					−0.48	H−1 → L
S6 (B_a)	B1U	6.99	177	0.3628	0.63	H−1 → L+1
			**Anthracene**			
State	Sym.	Energy (eV)	λ (nm)	Osc.strength	Wavefunction	
S1 (L_a)	B1U	3.99	311	0.1304	0.68	H → L
S2 (L_b)	B2U	4.22	294	0.0017	0.50	H−1 → L
					0.48	H → L+1
**S3**	**B3G**	**5.56**	**223**	**0**	**0.68**	**H−2 → L**
**S4**	**B3G**	**5.68**	**218**	**0**	**0.68**	**H → L+2**
**S5**	**AG**	**6.00**	**207**	**0**	**0.44**	**H−2 → L+1**
					0.40	H−1 → L+2
S6 (B_b)	B2U	6.03	206	3.0937	0.50	H → L+1
					−0.47	H−1 → L
**S7**	**B1U**	**6.32**	**196**	**0**	**0.54**	**H → L+3**
					**−0.46**	**H−3 → L**
**S8**	**B3U**	**6.60**	**188**	**0**	**0.68**	**H−5 → L**
S9 (B_a)	B1U	6.63	187	0.2023	0.63	H-1 → L+1

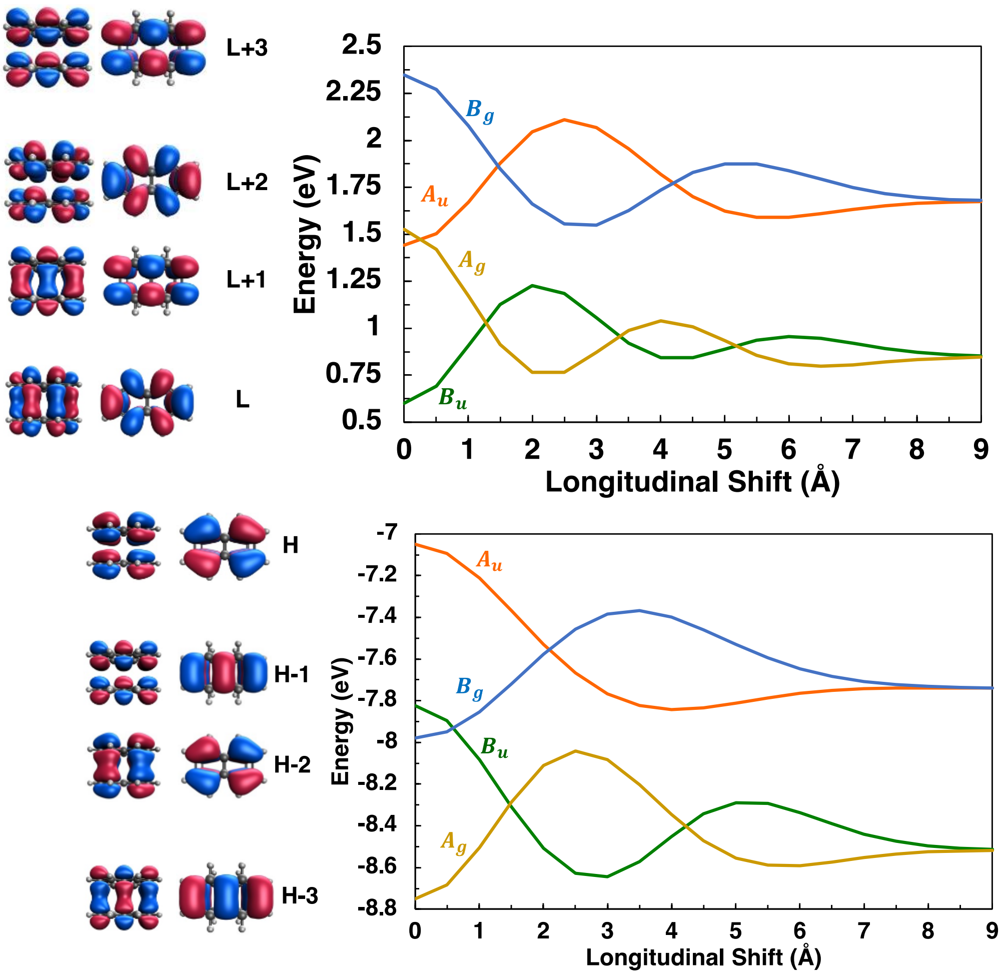

Figure S1. Energy profiles, as a function of the longitudinal translation coordinate, and symmetry of the frontier molecular orbitals of naphthalene included in the diabatization procedure. From ωB97X-D/6-31G* calculations.

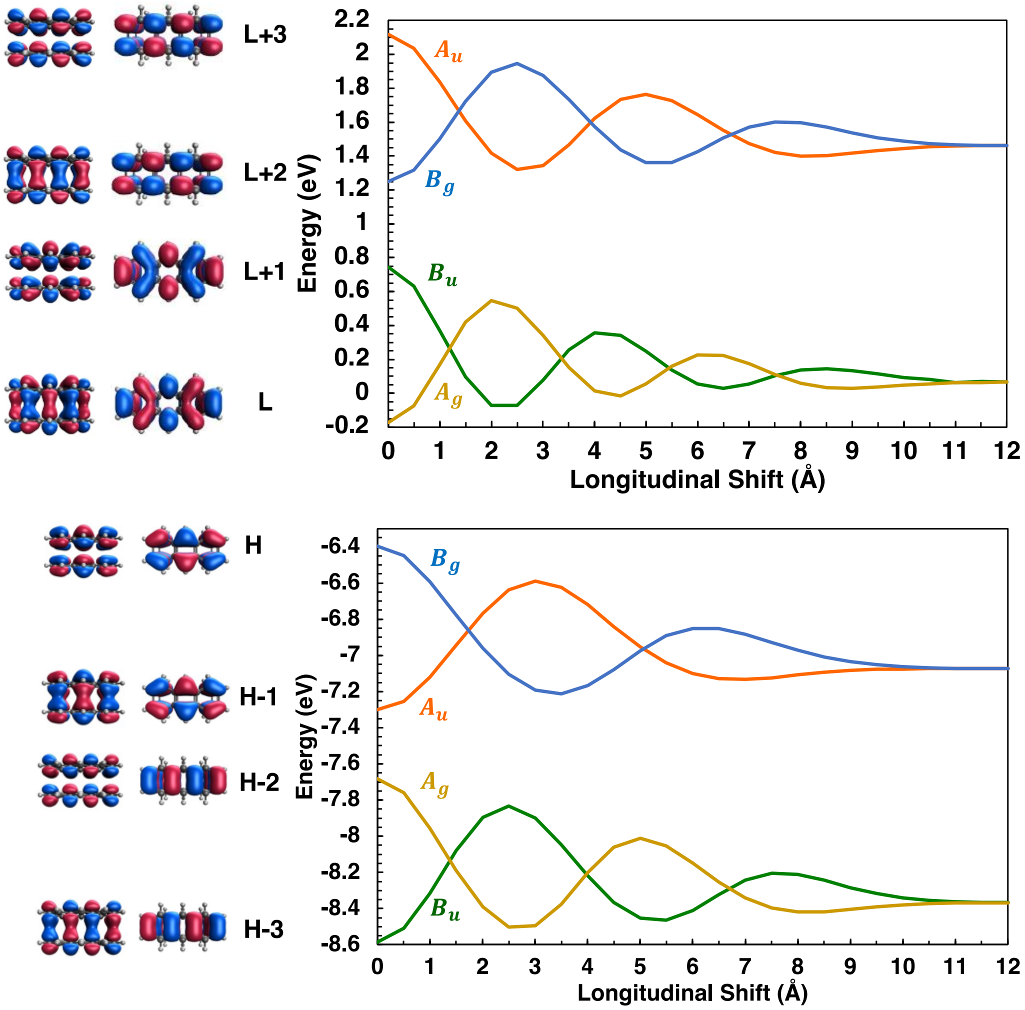

Figure S2. Energy profiles, as a function of the longitudinal translation coordinate, and symmetry of the frontier molecular orbitals of anthracene included in the diabatization procedure. From ωB97X-D/6-31G* calculations.

Table S3. Matrix elements of $\boldsymbol{H}_{dia}$ between diabatic states of the same type $(LE^{(n)}, CT^{(n)})$.

$\boldsymbol{H}_{dia}$	$LE_A^{(n)}$	$LE_B^{(n)}$	$CT_{AB}^{(n)}$	$CT_{BA}^{(n)}$
$LE_A^{(n)}$	$E_{LE}^{(n)}$	$V_e^{(n)}$	$D_e^{(n)}$	$D_h^{(n)}$
$LE_B^{(n)}$	$V_e^{(n)}$	$E_{LE}^{(n)}$	$D_h^{(n)}$	$D_e^{(n)}$
$CT_{AB}^{(n)}$	$D_e^{(n)}$	$D_h^{(n)}$	$E_{CT}^{(n)}$	$W^{(n)}$
$CT_{BA}^{(n)}$	$D_h^{(n)}$	$D_e^{(n)}$	$W^{(n)}$	$E_{CT}^{(n)}$

Table S4. Matrix elements of $\mathbf{H}_{\mathrm{dia}}$ between diabatic states of different types $(LE^{(n)}, CT^{(n)}$and $LE^{(p)}, CT^{(p)})$. The additional superscript $-p/-n$ for super-exchange interactions denotes the type of LE diabatic state.

$\boldsymbol{H}_{dia}$	$LE_A^{(p)}$	$LE_B^{(p)}$	$CT_{AB}^{(p)}$	$CT_{BA}^{(p)}$
$LE_A^{(n)}$	$H^{(n,p)}$	$V_e^{(n,p)}$	$D_e^{(n,p)-n}$	$D_h^{(n,p)-n}$
$LE_B^{(n)}$	$V_e^{(n,p)}$	$H^{(n,p)}$	$D_h^{(n,p)-n}$	$D_e^{(n,p)-n}$
$CT_{AB}^{(n)}$	$D_e^{(n,p)-p}$	$D_h^{(n,p)-p}$	$H_{CT}^{(n,p)}$	$W^{(n,p)}$
$CT_{BA}^{(n)}$	$D_h^{(n,p)-p}$	$D_e^{(n,p)-p}$	$W^{(n,p)}$	$H_{CT}^{(n,p)}$

Table S5. Matrix elements of $\boldsymbol{H}_{dia}^{SA}$ between SA diabatic states of $\boldsymbol{A_u}$ symmetry (+ combinations) and $\boldsymbol{B_g}$ symmetry (− combinations).

$\boldsymbol{H}_{dia}^{SA}$ $\boldsymbol{A_u}(+)/\boldsymbol{B_g}(-)$	$FE^{(1)\pm}$	$FE^{(3)\pm}$	$CR^{(1)\pm}$	$CR^{(3)\pm}$
$FE^{(1)\pm}$	$E(LE)^{(1)} \pm V_e^{(1)}$	$H^{(1,3)} \pm V_e^{(1,3)}$	$D_e^{(1)} \pm D_h^{(1)}$	$D_e^{(1,3)-1} \pm D_h^{(1,3)-1}$
$FE^{(3)\pm}$	$H^{(1,3)} \pm V_e^{(1,3)}$	$E(LE)^{(3)} \pm V_e^{(3)}$	$D_e^{(1,3)-3} \pm D_h^{(1,3)-3}$	$D_e^{(3)} \pm D_h^{(3)}$
$CR^{(1)\pm}$	$D_e^{(1)} \pm D_h^{(1)}$	$D_e^{(1,3)-3} \pm D_h^{(1,3)-3}$	$E(CT)^{(1)} \pm W^{(1)}$	$H_{CT}^{(1,3)} \pm W^{(1,3)}$
$CR^{(3)\pm}$	$D_e^{(1,3)-1} \pm D_h^{(1,3)-1}$	$D_e^{(3)} \pm D_h^{(3)}$	$H_{CT}^{(1,3)} \pm W^{(1,3)}$	$E(CT)^{(3)} \pm W^{(3)}$

Table S6. Matrix elements of $\boldsymbol{H}_{dia}^{SA}$ between SA diabatic states of $\boldsymbol{B_u}$ symmetry (+ combinations) and $\boldsymbol{A_g}$ symmetry (− combinations)

$\boldsymbol{H}_{dia}^{SA}$ $\boldsymbol{B_u}(+)/\boldsymbol{A_g}(-)$	$FE^{(2)\pm}$	$FE^{(4)\pm}$	$CR^{(2)\pm}$	$CR^{(4)\pm}$
$FE^{(2)\pm}$	$E(LE)^{(2)} \pm V_e^{(2)}$	$H^{(2,4)} \pm V_e^{(2,4)}$	$D_e^{(2)} \pm D_h^{(2)}$	$D_e^{(2,4)-2} \pm D_h^{(2,4)-2}$
$FE^{(4)\pm}$	$H^{(2,4)} \pm V_e^{(2,4)}$	$E(LE)^{(4)} \pm V_e^{(4)}$	$D_e^{(2,4)-4} \pm D_h^{(2,4)-4}$	$D_e^{(4)} \pm D_h^{(4)}$
$CR^{(2)\pm}$	$D_e^{(2)} \pm D_h^{(2)}$	$D_e^{(2,4)-4} \pm D_h^{(2,4)-4}$	$E(CT)^{(2)} \pm W^{(2)}$	$H_{CT}^{(2,4)} \pm W^{(2,4)}$
$CR^{(4)\pm}$	$D_e^{(2,4)-2} \pm D_h^{(2,4)-2}$	$D_e^{(4)} \pm D_h^{(4)}$	$H_{CT}^{(2,4)} \pm W^{(2,4)}$	$E(CT)^{(4)} \pm W^{(4)}$

NAPHTHALENE

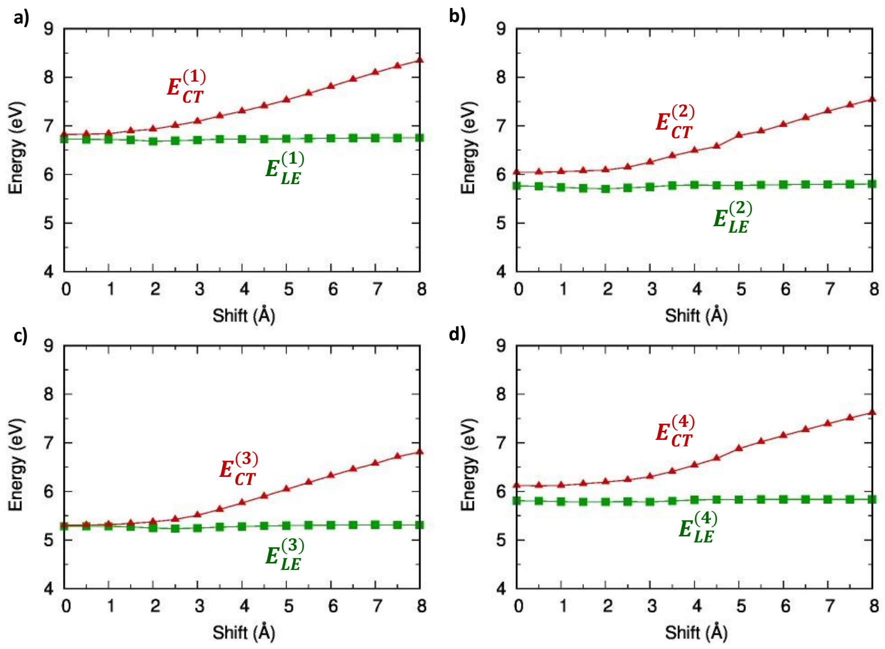

Figure S3. Energy profiles of the singlet diabatic states of naphthalene for the four types of diabatic states defined in eq.(1) of the manuscript

ANTHRACENE

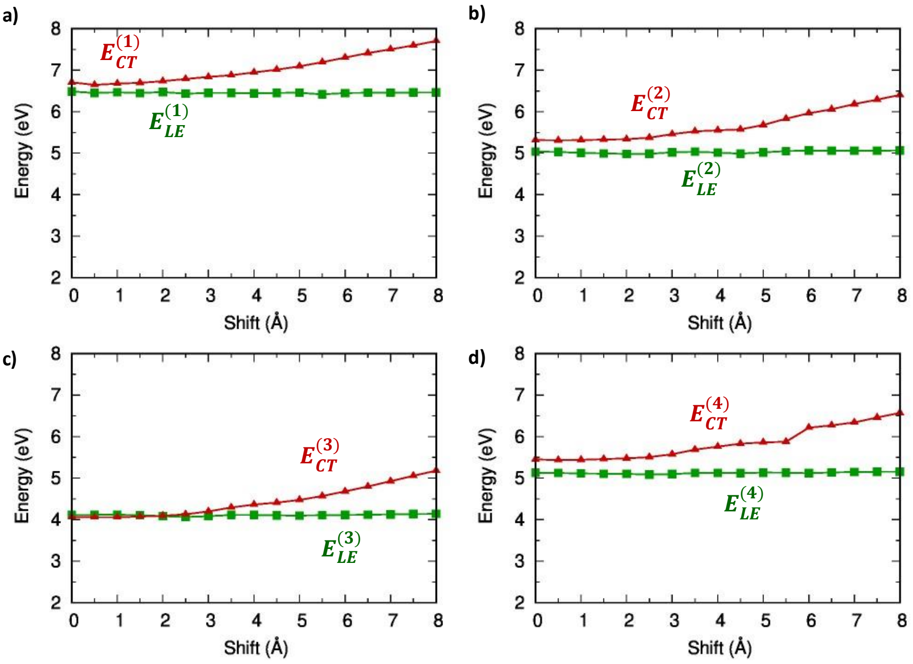

Figure S4. Energy profiles of the singlet diabatic states of anthracene for the four types of diabatic states defined in eq.(1) of the manuscript.

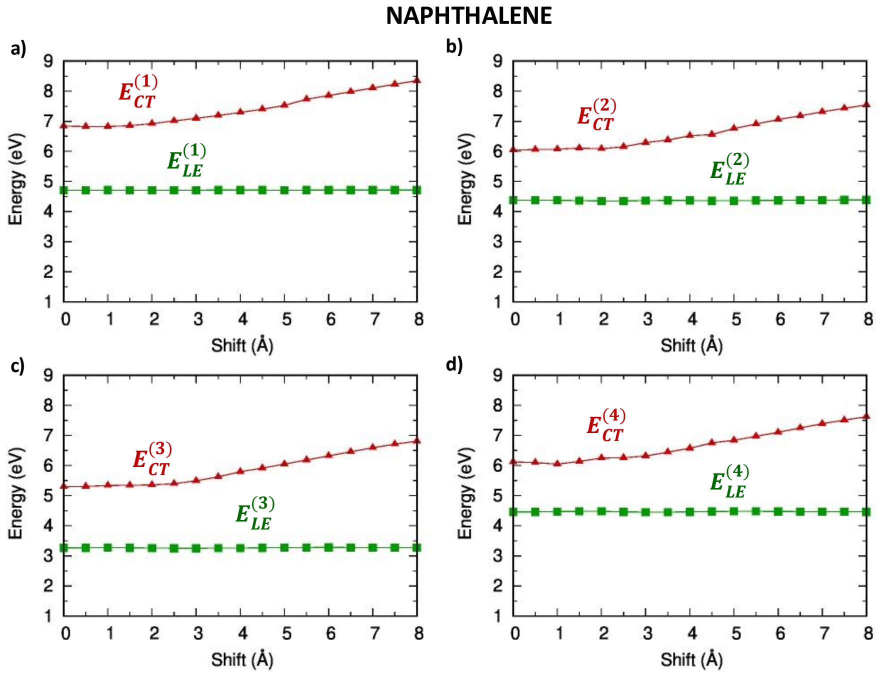

Figure S5. Energy profiles of the triplet diabatic states of naphthalene for the four types of diabatic states defined in eq.(1) of the manuscript.

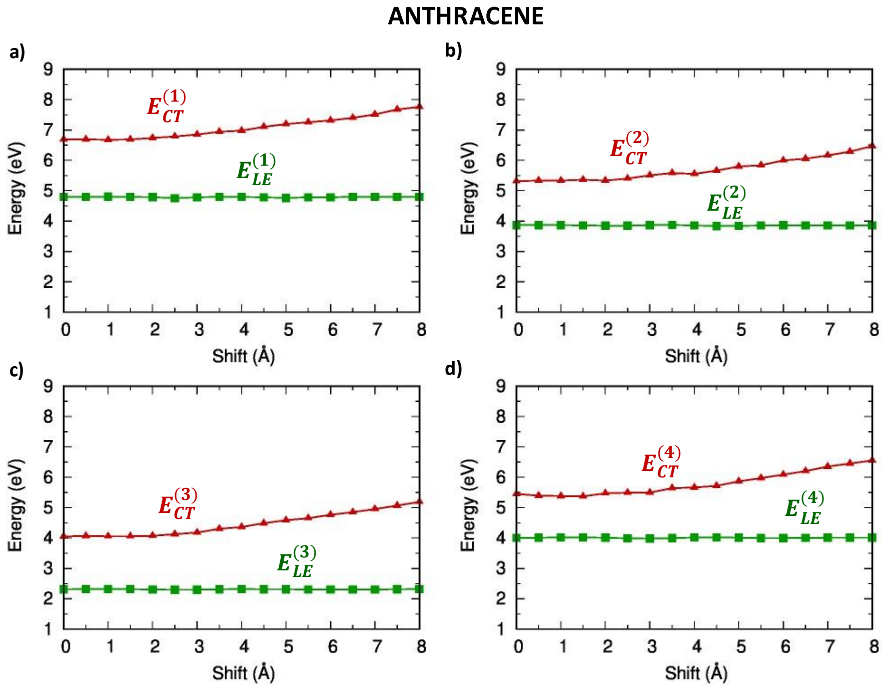

Figure S6. Energy profiles of the triplet diabatic states of anthracene for the four types of diabatic states defined in eq.(1) of the manuscript.

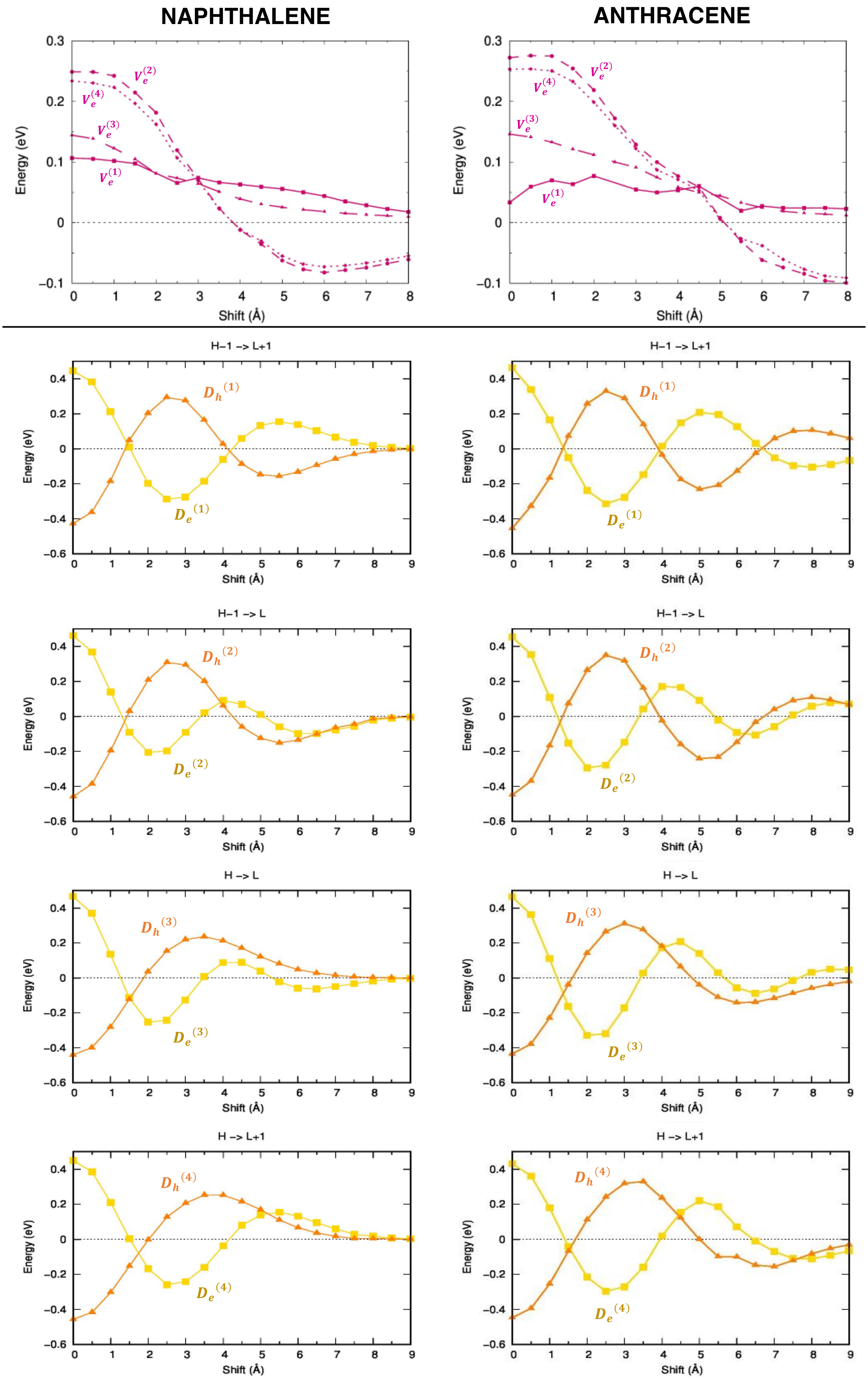

Figure S7. Energy profiles of the excitonic (top) and super-exchange (bottom) interactions between singlet diabatic states of the same type of (left) naphthalene and (right) anthracene.

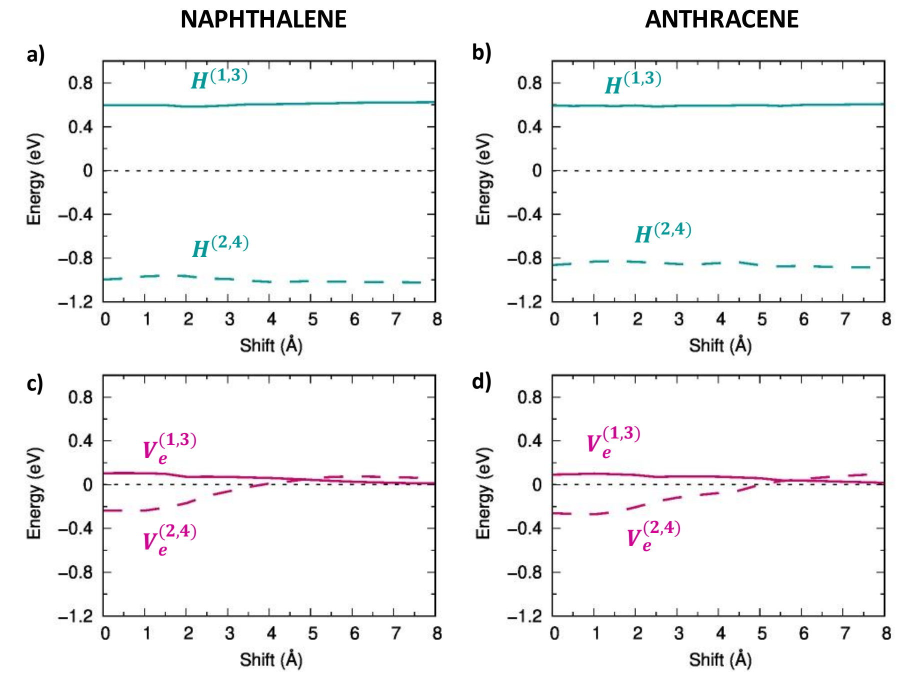

Figure S8. Energy profiles of interactions between LE diabatic singlet states of different type, along the longitudinal shift. (Top) Intramolecular interactions between LE diabatic states of (a) naphthalene and (b) anthracene dimers; (Bottom) Intermolecular interactions between LE diabatic states of different type of naphthalene (c) and anthracene (d).

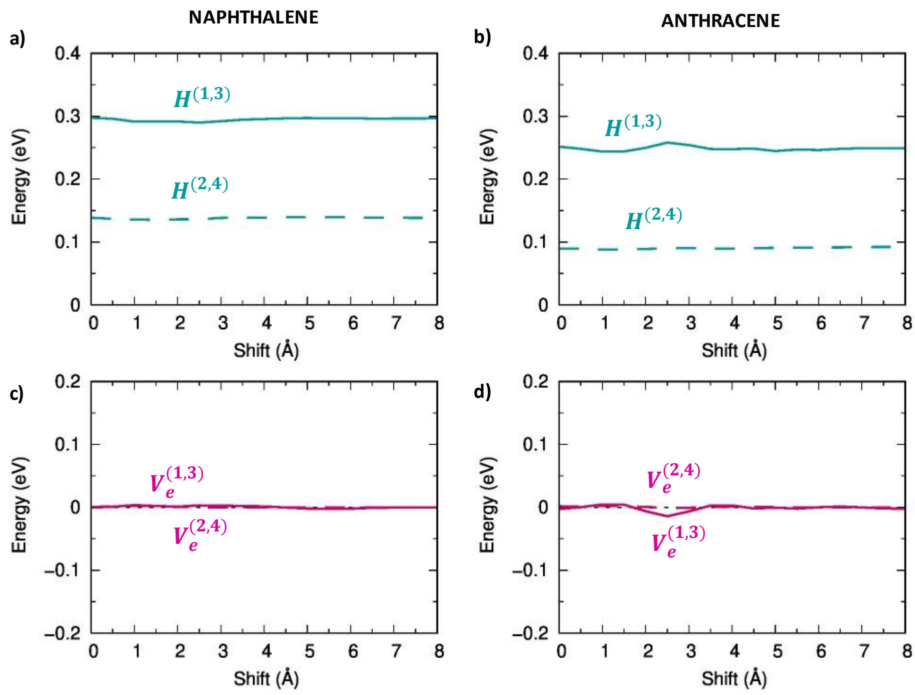

Figure S9. Energy profiles of interactions between LE diabatic triplet states of different type, along the longitudinal shift. (Top) Intramolecular interactions between LE diabatic states of (a) naphthalene and (b) anthracene dimers; (Bottom) Intermolecular interactions between LE diabatic states of different type of naphthalene (c) and anthracene (d).

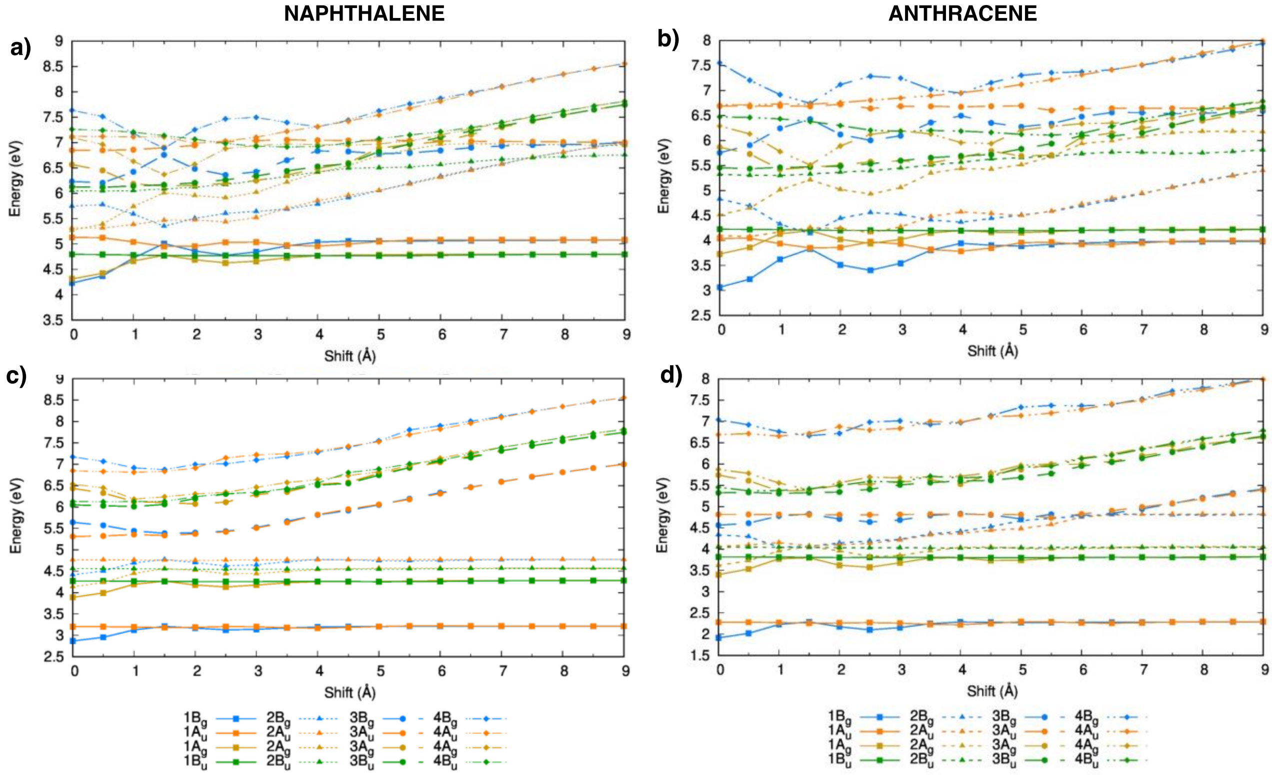

Figure S10. Adiabatic excitation energy profiles along the longitudinal shift of the set of 16 singlet (a,b) and triplet (c, d) exciton states analysed with the diabatization procedure. Naphthalene (left) and anthracene (right). From TDA-ωB97X-D /6-31G* calculations.

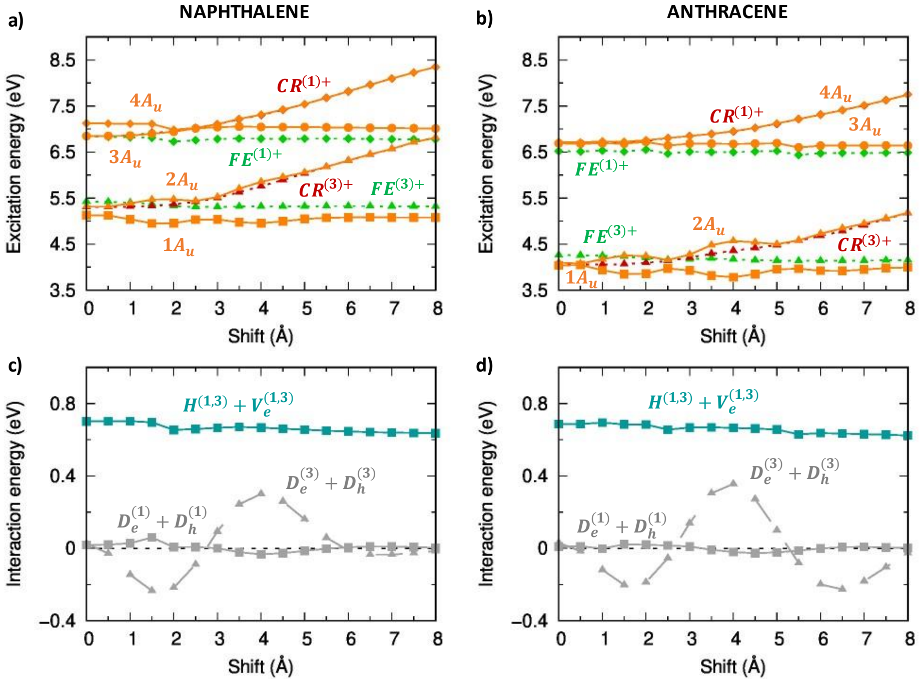

Figure S11. Analysis of the excitation energy profiles of singlet exciton states (A_u symmetry) for (left) naphthalene and (right) anthracene dimer (TDA-ωB97X-D/6-31G*) in terms of SA diabatic states (green for FE states, red for CR states) and their interactions. (a, b) Computed adiabatic and SA diabatic excitation energy profiles. (c, d) Magnitude and modulation along the longitudinal translation coordinate of the (grey) $D_e^{(1)} + D_h^{(1)}$, $D_e^{(3)} + D_h^{(3)}$ interactions, coupling FE and CR states, and of the (dark-turquoise) $H^{(1,3)} + V_e^{(1,3)}$ interactions mixing $FE^{(1)}$ and $FE^{(3)}$ states.

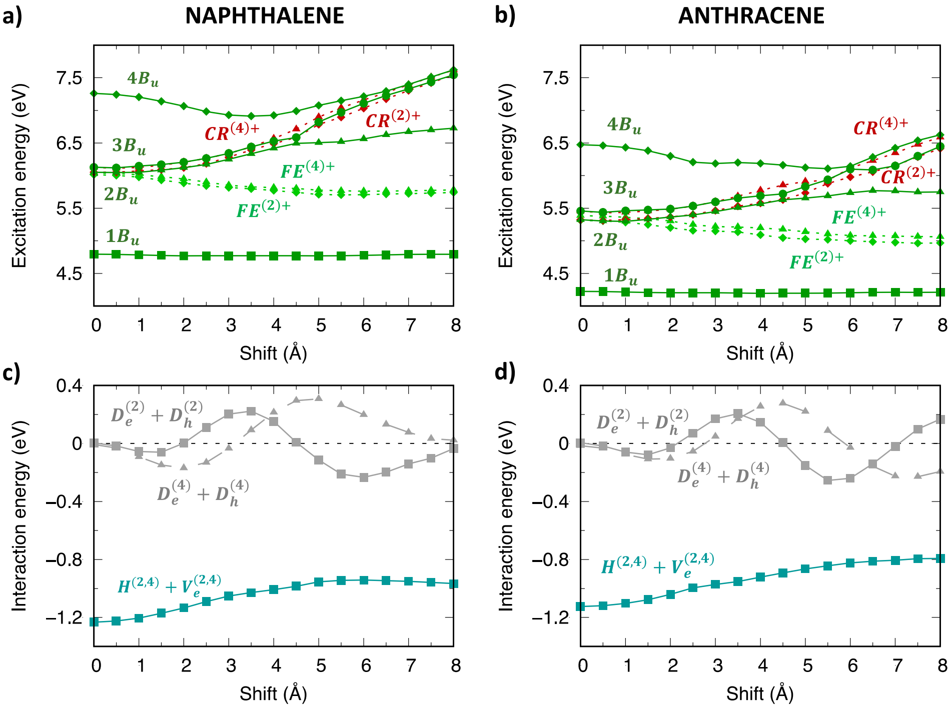

Figure S12. Analysis of the excitation energy profiles of singlet exciton states (B_u symmetry) for (left) naphthalene and (right) anthracene dimer (TDA-ωB97X-D/6-31G*) in terms of SA diabatic states (green for FE states, red for CR states) and their interactions. (a,b) Computed adiabatic and SA diabatic excitation energy profiles. (c, d) Magnitude and modulation along the longitudinal translation coordinate of the (grey) $D_e^{(2)} + D_h^{(2)}$, $D_e^{(4)} + D_h^{(4)}$ interactions, coupling FE and CR states, and of the (dark-turquoise) $H^{(2,4)} + V_e^{(2,4)}$ interactions mixing $FE^{(2)}$ and $FE^{(4)}$ states.

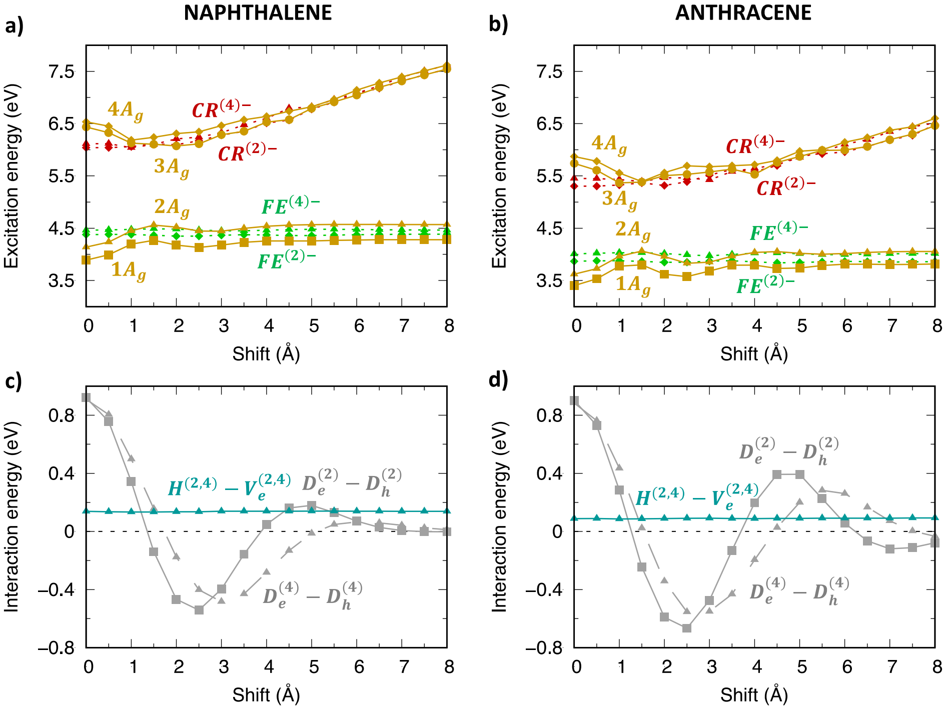

Figure S13. Analysis of the excitation energy profiles of triplet exciton states (A_g symmetry) for (left) naphthalene and (right) anthracene dimer (TDA-ωB97X-D/6-31G*) in terms of SA diabatic states (green for FE states, red for CR states) and their interactions. (top) Computed adiabatic and SA diabatic excitation energy profiles. (bottom) Magnitude and modulation along the longitudinal translation coordinate of the (grey) $D_e^{(2)} - D_h^{(2)}$, $D_e^{(4)} - D_h^{(4)}$ interactions, coupling FE and CR states, and of the (dark-turquoise) $H^{(2,4)} - V_e^{(2,4)}$ interactions mixing $FE^{(2)}$ and $FE^{(4)}$ states.

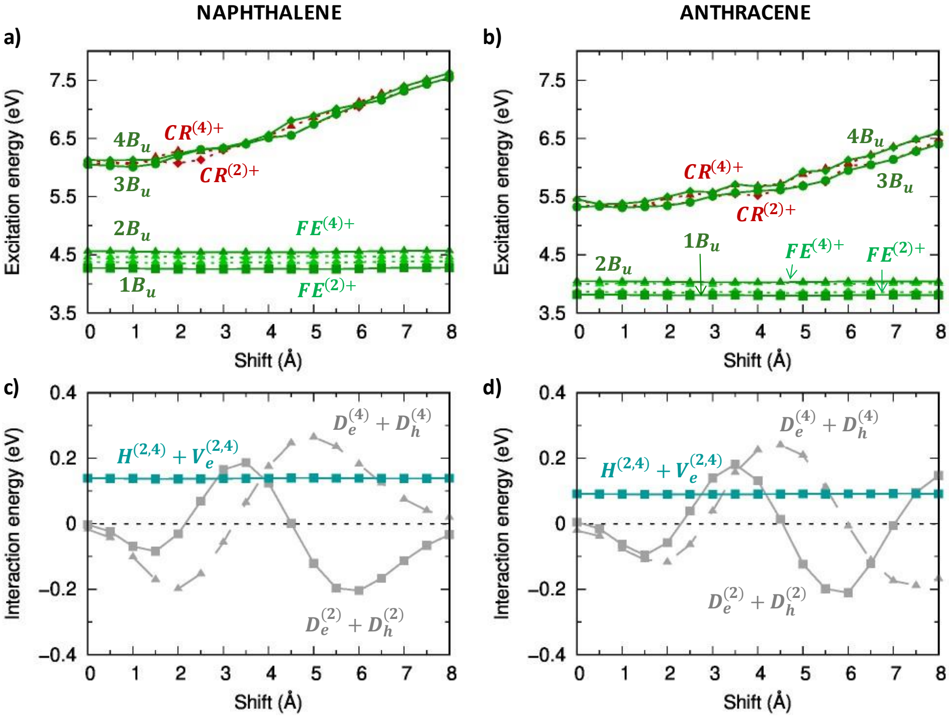

Figure S14. Analysis of the excitation energy profiles of triplet exciton states (B_u symmetry) for (left) naphthalene and (right) anthracene dimer (TDA-ωB97X-D/6-31G*) in terms of SA diabatic states (green for FE states, red for CR states) and their interactions. (top) Computed adiabatic and SA diabatic excitation energy profiles. (bottom) Magnitude and modulation along the longitudinal translation coordinate of the (grey) $D_e^{(2)} + D_h^{(2)}$, $D_e^{(4)} + D_h^{(4)}$ interactions, coupling FE and CR states, and of the (dark-turquoise) $H^{(2,4)} + V_e^{(2,4)}$ interactions mixing $FE^{(2)}$ and $FE^{(4)}$ states.

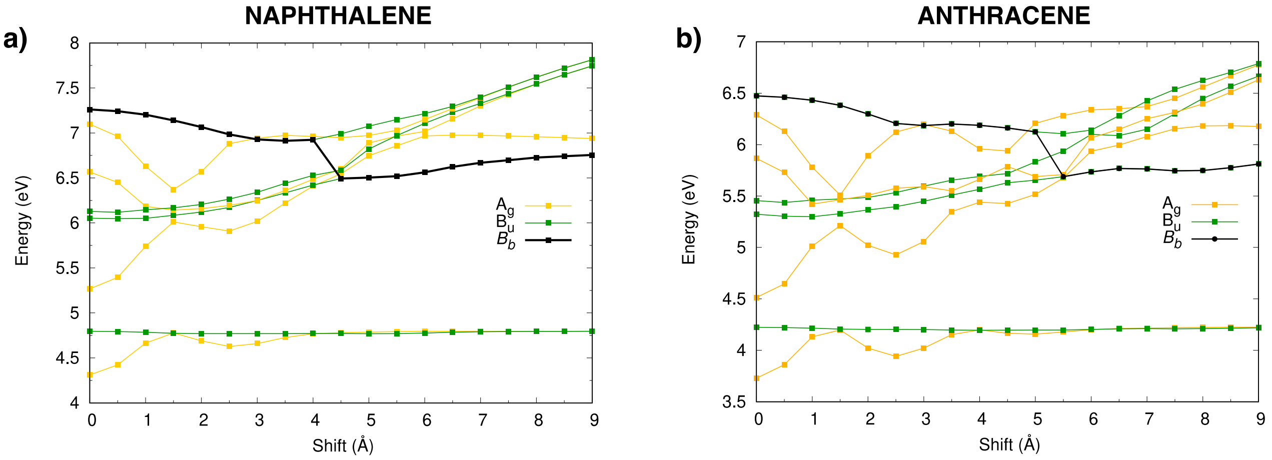

Figure S15. Excitation energy profiles of the singlet adiabatic exciton state of A_g (yellow) and B_u (green) symmetry of (a) naphthalene and (b) anthracene. The evolution of the strongly allowed exciton state (state that bears a parentage with monomers' B_b state) is shown in black. TDA-ωB97X-D/6-31G* calculations.

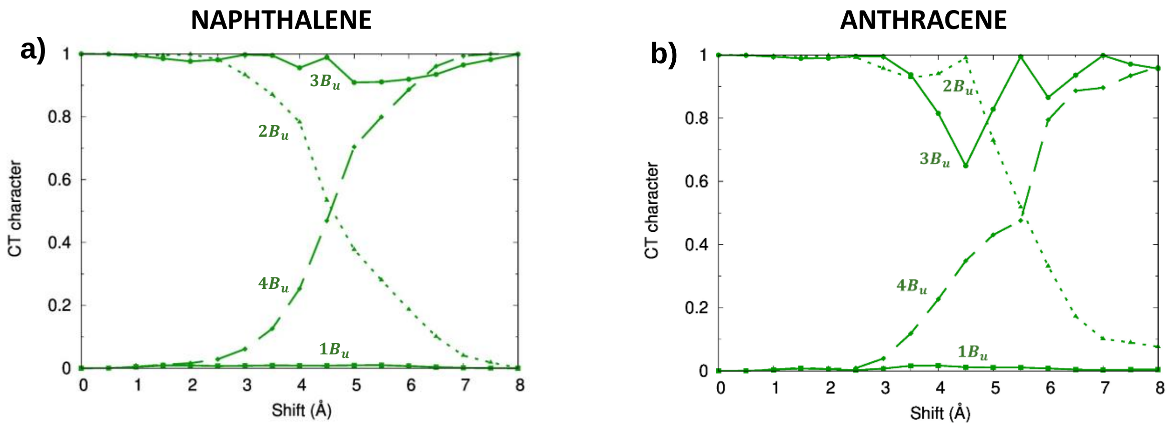

Figure S16. CT character of the singlet adiabatic exciton states of B_u symmetry along the longitudinal translation coordinate for (a) naphthalene and (b) anthracene.

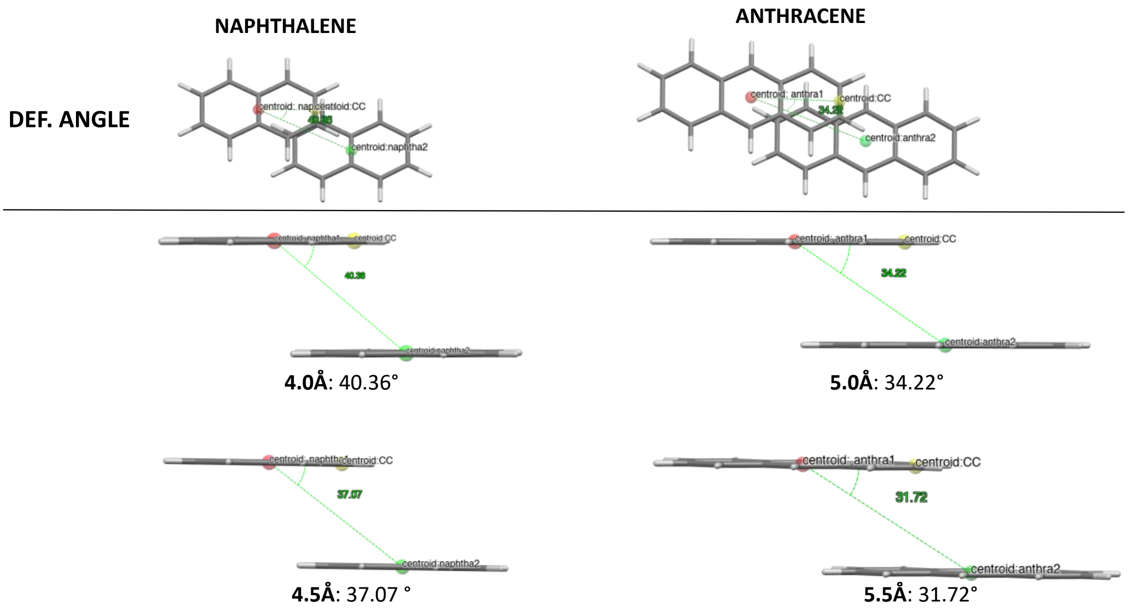

Figure S17. Interplanar angles (*i.e.* angle in-between transition dipole moments of each monomer) of naphthalene (left) and anthracene (right) dimers. (Top) Definition of the angle. (Bottom) Angle values reported for 4.0Å , 4.5Å displaced naphthalene and for 5.0Å, 5.5Å displaced anthracene. As mentioned in the main paper, these displacements correspond, respectively, to the region of H-/J-aggregate exchange.
